# Tissue factor is an angiogenic-specific receptor for factor VII-targeted immunotherapy and photodynamic therapy

**DOI:** 10.1007/s10456-016-9530-9

**Published:** 2016-11-02

**Authors:** Zhiwei Hu, Jijun Cheng, Jie Xu, Wolfram Ruf, Charles J. Lockwood

**Affiliations:** 1 Department of Surgery Division of Surgical Oncology, The James Comprehensive Cancer Center (OSUCCC), The Ohio State University College of Medicine, Columbus, OH 43210 USA; 2 Department of Obstetrics, Gynecology and Reproductive Sciences, Yale University School of Medicine, New Haven, CT 06520 USA; 3 Department of Immunology and Microbial Science, The Scripps Research Institute, La Jolla, CA 92037 USA; 4 Department of Obstetrics and Gynecology, Morsani College of Medicine, University of South Florida, Tampa, FL 33612 USA; 5 Department of Genetics, Yale University, New Haven, CT USA; 6 Institute of Cancer Stem Cell, Dalian Medical University, Dalian, China

**Keywords:** Angiogenesis, VEGF, Tissue factor, Factor VII-targeted therapy, Vascular endothelial cells

## Abstract

Identification of target molecules specific for angiogenic vascular endothelial cells (VEC), the inner layer of pathological neovasculature, is critical for discovery and development of neovascular-targeting therapy for angiogenesis-dependent human diseases, notably cancer, macular degeneration and endometriosis, in which vascular endothelial growth factor (VEGF) plays a central pathophysiological role. Using VEGF-stimulated vascular endothelial cells (VECs) isolated from microvessels, venous and arterial blood vessels as in vitro angiogenic models and unstimulated VECs as a quiescent VEC model, we examined the expression of tissue factor (TF), a membrane-bound receptor on the angiogenic VEC models compared with quiescent VEC controls. We found that TF is specifically expressed on angiogenic VECs in a time-dependent manner in microvessels, venous and arterial vessels. TF-targeted therapeutic agents, including factor VII (fVII)-IgG1 Fc and fVII-conjugated photosensitizer, can selectively bind angiogenic VECs, but not the quiescent VECs. Moreover, fVII-targeted photodynamic therapy can selectively and completely eradicate angiogenic VECs. We conclude that TF is an angiogenic-specific receptor and the target molecule for fVII-targeted therapeutics. This study supports clinical trials of TF-targeted therapeutics for the treatment of angiogenesis-dependent diseases such as cancer, macular degeneration and endometriosis.

## Introduction

Angiogenesis, the formation of new blood vessels from existing blood vessels, is a common and critical pathological process in many human diseases [1], including but not limited to cancer, age-related macular degeneration (AMD), endometriosis and rheumatoid arthritis. For example, in cancer, the newly formed blood vessels, usually termed as tumor angiogenic vessels or tumor neovasculature, can be differentiated from normal (resting, quiescent) vessels in normal tissues in morphology (vascular and endothelial shapes), and function (increased permeability) [2, 3]. As such, tumor neovasculature provides not only nutrients and oxygen for cancer cells to proliferate, but also serves as a conduit for cancer cells to metastasize. Thus, the tumor neovasculature is a key target for development of therapeutic agents. The search for biomarkers as target molecules specific for angiogenic vascular endothelial cells (VEC), the inner layer of pathological neovasculature, is promising and critical in developing novel neovascular-targeting therapies for these common human diseases [1].

One potential approach to eliminating pathological neovascularization involves attacking the subset of vascular endothelial cells that are angiogenic, via a cell-type-specific surface molecule coupled to a cell-killing agent. Based on the in vivo observation of TF expression on tumor vascular endothelial cells in breast cancer tissues from patients [4] and some mouse models of human tumor xenografts [5–7], we have begun developing such an approach using tissue factor (TF), a 47-kDa membrane-bound receptor [4, 8–11], as the target molecule on angiogenic VECs. To assemble this TF-directed treatment, we used, as the starting molecule, the principal ligand of TF, factor VII (fVII) [12]. To eliminate the coagulation activity of zymogenic fVII, a coagulation active site lysine 341 was replaced by an alanine (thereafter containing the mutation of K341A in fVII polypeptides unless specified) using site-directed mutagenesis [5, 13, 14]. Using this strategy, we have pursued two different therapeutic approaches; immunotherapy (by fusing fVII to an IgG1 Fc as an antibody-like immunoconjugate, i.e., fVII/IgG1 Fc, also called ICON) [5, 14] and photodynamic therapy (PDT) (by chemically conjugating fVII peptides with photosensitizers (PS), fVII/PS, for fVII-targeted PDT, abbreviated as fVII-tPDT) [15, 16] for the treatment of cancers including melanoma [5, 13], prostate [14], head and neck [17], breast [7, 15, 16] and lung [6], as well as age-related macular degeneration (AMD) [18–20] and endometriosis [21] in preclinical animal models and in phase I and II clinical trials for AMD patients (NCT01485588) [22]. In vivo data confirm that the TF-targeted agents ICON and fVII-tPDT can selectively eradicate pathological neovasculature in cancer [13, 23], AMD [18–20] and endometriosis [21]. However, it remains unclear which angiogenic growth factor induces TF expression commonly detected in these pathological angiogenesis-dependent malignancy and non-malignant diseases, whether TF is also selectively expressed by in vitro angiogenic VEC models and whether TF-targeted agents can selectively eradicate in vitro models of angiogenic VECs via targeting TF without harming quiescent VECs. This study is designed to address these questions by identifying a common angiogenic growth factor (for example, VEGF) in these angiogenic-dependent diseases, examining TF expression on in vitro models of angiogenic and quiescent VECs and then testing the selectivity and effectiveness of TF-targeted agents (ICON and fVII-PS) for binding and killing the in vitro angiogenic VECs.

Vascular endothelial growth factor (VEGF) plays a central role in angiogenesis-dependent cancer and non-malignant human diseases [24], such as macular degeneration [25], rheumatoid arthritis [26] and endometriosis [27]. Specifically, VEGF stimulates angiogenesis by binding to VEGR receptors on VECs in the pathological neovasculature (usually micro- or capillary vessels) in those angiogenesis-dependent diseases. In humans, there are three major types of blood vessels, namely micro- or capillary vessels, veins and arteries. Accordingly, VEC, the inner layer of blood vessels, can be isolated from microvessels, venous and arterial vessels. It is previously known that VEGF can induce TF expression on human umbilical vein endothelial cells (HUVEC), a commonly used VEC model in angiogenesis studies. To better mimic pathological angiogenesis, an ideal angiogenic VEC model should be derived from micro- or capillary vessels. This study is designed to investigate whether angiogenic VECs from micro- or capillary vessels express TF upon VEGF stimulation and whether fVII-dependent therapies target non-pathological (quiescent) endothelial cells using VEGF-stimulated and unstimulated VECs as in vitro models. Therefore, in the current study, we examined the expression patterns of TF on in vitro models of angiogenic and quiescent VECs from three types of blood vessels and sought to rigorously assess the validity of the claim that dimeric fVII-IgG1Fc (ICON) in immunotherapy and monomer fVII peptide in PDT are selective and effective in killing angiogenic VECs via TF targeting.

## Materials and methods

### Cell culture

HMVECs (Lonza), HUVEC (generously provided by the Yale Vascular Biology and Therapeutics (VBT) Program) and HAEC (Lonza) were purchased and grown in growth medium supplemented with heat-inactivated fetal bovine serum (FBS, Sigma) and 1× penicillin and streptomycin (Gibco), following the manufacturers’ protocols. Lung-derived normal human microvascular endothelial cells (HMVEC-LBI, simplified as HMVEC) were purchased (Lonza). As assayed by the manufacturer, the HMVEC cells were endothelial marker CD31 positive, lymphatic endothelial marker podoplanin negative and muscle cell marker alpha actin negative. These assay results confirmed their origin of blood VEC. It was cultured in Lonza’s growth medium, EBM-2 basal medium supplemented with EGM-2 MV SingleQuot Kit supplements and growth factors (Lonza) and 20% FBS, propagated with the ReagentPack Subculture Reagents (Lonza) containing the three solutions, trypsin/EDTA, trypsin neutralizing solution, and HEPES-buffered saline. HUVECs were grown in M199 growth medium supplemented with 20% heat-inactivated FBS (HI-FBS) and 1:100 ECGS solution, as described [15]. HAECs (Lonza) were grown in EGM-2 medium supplemented with 10% HI-FBS. Cells are cultured at 37 °C with 5% CO_2_. Human breast cancer MDA-MB-231 line was grown in DMEM supplemented with 10% FBS. CHO-K1 lines were grown in F-12 K (ATCC) medium with 10% FBS and 0.5 mg/ml G418 (Invitrogen), including CHO-K1 cell lines stably expressing TF, EPCR or TF + EPCR. Untransfected CHO-K1 cells were used as negative control for TF and EPCR and for generating a stable CHO-K1/EPCR line by transfection with a plasmid encoding EPCR (kind gift from Dr. Ruf).

### Generation of in vitro angiogenic and quiescent VEC models by VEGF stimulation

HMVECs (Lonza), HUVEC (Yale VBT group) and HAEC (Lonza) were first seeded with 5 × 10^3^ cells/cm^2^ cultured in complete growth media with heat-inactivated FBS and growth factors, either in 6-well microplates (1.25 × 10^5^ cells per well for extraction of cell lysates for Western blotting), 96-well microplates (1.5 × 10^3^ cells in 100 µl per well for cell ELISA and fVII-tPDT) or in 8-chamber slides (3.5 × 10^3^ cells in 500 µl per chamber for immunofluorescent staining and confocal microscopy), until 80–90% confluence is reached. Prior to VEGF stimulation, the VECs were washed ×3 with HEPES-buffered saline to remove trace of FBS and growth factors (to minimize their potential effect on induction of TF and EPCR expression) and then starved in serum-free and growth factors-free EBM-2 basal medium (Lonza) or endothelial cell serum-free medium (Invitrogen) overnight. The cells were then exchanged with half of the same serum-free medium supplemented with a final concentration of 1 nM VEGF (BD Biosciences), a concentration that was previously tested for induction of TF expression on HUVEC [28], for 2, 4, 6, 8 and 24 h as angiogenic VEC models. The VECs were starved but were not stimulated with VEGF as quiescent VEC control (0 h). And then the VECs were used as in vitro angiogenic (1 nM VEGF stimulation for 4 h as TF reaching the peak expression at this time point) and quiescent VEC models in experiments with Western blotting, confocal microscopy, cell ELISA and fVII-tPDT treatment and mechanism studies.

### Immunofluorescent staining and confocal microscopic imaging

For confocal microscopy, the VECs grown on chamber glass slides were fixed with 4% paraformaldehyde (PFA) and then were immunostained for TF using mouse monoclonal antihuman TF (HTF1) (kind gift from William Konigsberg) (clone HTF 1) [29], antihuman endothelial marker CD31 PE conjugate (BD Biosciences) and nuclei staining dye DAPI (Molecular Probes) for confocal microscopy.

### Protein samples of angiogenic and quiescent VECs for Western blotting

For Western blotting, protein samples were extracted from the VECs (HMVEC, HUVEC and HAEC) by TRIzol reagent (Invitrogen) followed by immunoblotting for TF and GAPDH expression using antihuman TF (HTF1) [29] and anti-GAPDH (Research Diagnostic Inc) antibodies, and the band intensities were normalizing to GAPDH using NIH ImageJ analysis.

### Cell ELISA and inhibitory cell ELISA

For cell ELISA, the VECs grown in 96-well plates were assayed for binding of mfVII/hIgG1 Fc, mfVII/Sp and anti-TF (HTF1) antibodies followed by anti-mfVII (for mfVII proteins) and then by corresponding secondary antibody HRP conjugates (antihuman IgG, anti-mouse IgG or anti-rat IgG antibodies). For inhibitory cell ELISA, the VECs were incubated first with anti-HTF or anti-EPCR antibodies (clone RCR-379, Sigma, as control antibody) prior to incubation with mfVII/hIgG1 Fc at molar ratios from 0.1:1 to 10:1 (antibody to fVII/IgG1 Fc).

We compared two anti-HTF antibodies, mouse monoclonal antihuman TF (HTF1) and goat polyclonal antihuman TF (goat anti-TF, American Diagnostica), for their inhibitory effect on mfVII/IgG1 Fc binding to human breast cancer MBA-MB-231 cell line, which was known to express a high level of TF [15, 16]. For fVII blocking experiments, we first stimulated HMVEC with 1 nM VEGF for 4 h then fixed the cells. After blocking with 1% BSA, we incubated the VEGF-stimulated HMVEC with goat anti-TF (American Diagnostica) or mouse anti-TF (HTF1) antibodies then added mfVII/hIg Fc. To determine whether fVII is a ligand for TF, we developed an inhibitory cell ELISA assay, in which angiogenic VEC (stimulated with 1 nM VEGF for 4 h) was first fixed by 4% paraformaldehyde (PFA) and subsequently blocked by 0.1–100 nM of inhibitory anti-HTF (goat anti-HTF) or anti-EPCR (RCR-379), which blocks binding of EPCR ligand to EPCR [30]. The procedure was terminated by incubation with 10 nM mfVII/hIgG Fc (molar ratios 0.01:1–10:1, inhibitory antibody to mfVII/hIgG Fc) and then with anti-hIgG HRP conjugate.

### Production of mfVII/Sp- or mfVII/NLS-SnCe6 conjugates

The procedures have been described in detail [6, 15, 16]. Briefly, the mfVII/Sp protein is composed of murine fVII(K341A), an S peptide (Sp) tag with a mutation at D14 N and a polyhistidine tag (His tag) for protein purification and detection (mfVII(K341A)/Sp(D14 N)/His, abbreviated as fVII/Sp [15, 16] for simplification). The mfVII/NLS protein is composed of the coding sequences for mfVII(K341A), 2 repeats of the wild-type nuclear localization sequence (NLS) (PKKKRKVG) of SV40 T-antigen [31], a tyrosine residue (requisite for future radioiodination imaging) and a His tag (mfVII(K341A)/NLS/His, abbreviated as fVII/NLS). The fVII/Sp and fVII/NLS proteins were produced using CHO-K1 producer cells and purified using the Ni-NTA affinity resin as described [15, 16]. The procedure for the conjugation of mfVII proteins to photosensitizer SnCe6 was described [15, 16].

### In vitro fVII-tPDT for HMVECs

For fVII-tPDT, HMVECs grown in 96-well plates were incubated with 2 μM fVII/Sp-SnCe6 or fVII/NLS-SnCe6 for 90 min, which was previously optimized as the drug–light interval for fVII-tPDT [16], then were irradiated (635 nm, 36 J/cm^2^) with a 635-nm fiber-coupled diode laser (BWF2-635-0.1-100-0.22, B&W Tek, Inc.). The fluorescence rate of the laser unit was measured by using a laser power meter (LaserCheck, Coherent, Inc.) prior to carrying out PDT. We used two assays to determine the effect of fVII-tPDT, one was crystal violet assay staining for monolayer membrane loss (overnight after fVII-tPDT) and the other was clonogenic assay for observing longer-term viability (10-14 days after fVII-tPDT), as described [7, 15, 16, 32]. Briefly, HMVEC cells, uninduced or induced by VEGF for 4 h, were washed with 1× HBSS containing 1% BSA, incubated with different concentrations of SnCe6-conjugated mfVII/Sp or mfVII/NLS, or free SnCe6 in the incubation buffer (1× HBSS with 1% BSA and 10 mM CaCl_2_), or no treatment in the buffer as controls, and cultured for 90 min at 37 °C. Then, the cells were washed and refilled with the growth medium for laser irradiation at 635 nm for 36 J/cm^2^.

### Crystal violet staining and clonogenic assay

Crystal violet staining and clonogenic assays after PDT were performed as described [15, 16]. Briefly for clonogenic assay, HMVECs in 96-well plates after PDT were cultured overnight, were transferred to a 60-mm dish and were diluted at tenfold serial dilutions. The cell suspensions were cultured for 12–14 days to allow colony formation, followed by fixation and crystal violet staining at the same condition used above for staining cells directly after PDT. Visible colonies of more than 50 cells were counted, and total cells in each original well, designated as colony formation units # (CFU), were back calculated by colony number times dilution factor. Percent colony formation, in comparison with no-treatment controls, was calculated as (colony # in experimental wells−average colony # in no-treatment wells) × 100%.

### Apoptosis and necrosis assays for mechanisms of in vitro PDT

Cells cultured in 96-well plate after PDT were immediately washed with the HEPES buffer (10 mM HEPES, pH 7.4, 140 mM saline, 2.5 mM CaCl_2_), stained with 1:20 Annexin V-FITC (Invitrogen), for phosphastidylserine, and 1 μg/ml of propidium iodide (PI), for DNA, in the HEPES buffer for 15 min at room temperature, and then washed and refilled with in 1× DPBS. The cells were visualized and photographed immediately under a fluorescent microscope.

### Statistical analyses

All quantitative assays were done in duplicates to calculate averages and standard deviation (mean ± SD). The half maximal effective concentrations (EC_50_) of fVII-tPDT and ntPDT were calculated by the best-fit linear regression or nonlinear one-phase decay equations using Prism software (GraphPad). *P* values were calculated using 2-way ANOVA with multiple comparisons test using Prism software (GraphPad), as specified in each figure legend.

## Results

To test for expression of angiogenic surface receptors, we used VEGF to generate angiogenic VEC models in vitro, and tested for the presence of TF on primary human VECs derived from three major types of mammalian vessels: (1) HMVEC, human microvascular endothelial cells; (2) HUVEC, human umbilical venous endothelial cells; and (3) HAEC, human aortic endothelial cells.

**Fig. 1:**
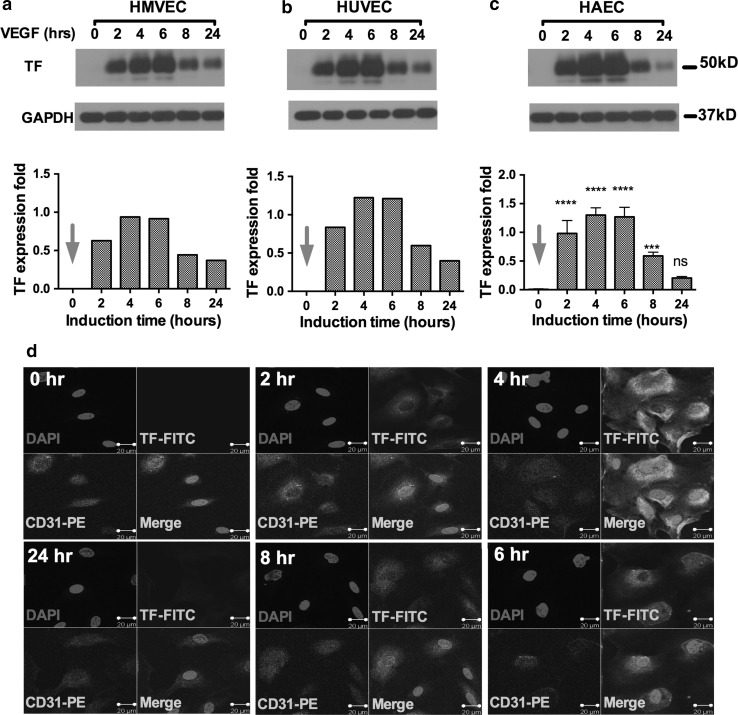
TF is an angiogenic-specific receptor on endothelial cells. **a**–**c**. Representative imaging of Western blots and expression fold changes (normalized to GAPDH) for TF in HMVEC (**a**), HUVEC (**b**) and HAEC (**c**) before (0 h) and after VEGF stimulation (2–24 h). **d** Representative confocal imaging of TF (*green*) and endothelial marker CD31 (*red*) expression on HMVEC before (0 h) and after VEGF stimulation (2–24 h). Cell nuclei were counterstained by DAPI (*blue*). *Scale bars*: 20 μm. *p* values were calculated by 2-way ANOVA with multiple comparisons test. Data in **a–c** are presented as Mean ± SD and representative of two independent experiments. (Color figure online)

We first examined the time course of TF expression on angiogenic and quiescent HMVEC (Fig. 1a), HUVEC (Fig. 1b) and HAEC (Fig. 1c). The Western blotting results in Fig. 1 showed that (1) TF protein, reported MW 47 kDa, shown in Fig. 1 as a band at ~50 kDa, was not detected in quiescent VEC (starved and unstimulated) (time point = 0 h, red arrows in TF expression fold chart) from all three VECs; (2) TF was detected in VEGF-treated angiogenic VECs (HMVEC, HUVEC and HAEC) within 2 h of VEGF stimulation; (3) and peaked at 4–6 h, then started decreasing at 6 h post-stimulation.

To confirm the time course of induced TF expression on endothelial cells, we stained VEC for TF and the endothelial marker CD31 and observed the stained VEC under confocal microscope. The results in Fig. 1d showed that TF was not detected on quiescent VEC (0 h). After VEGF stimulation, however, TF expression was detected at 2 h with a peak in fluorescent intensity at 4–6 h followed by a decrease that was still visible at 24 h.

**Fig. 2:**
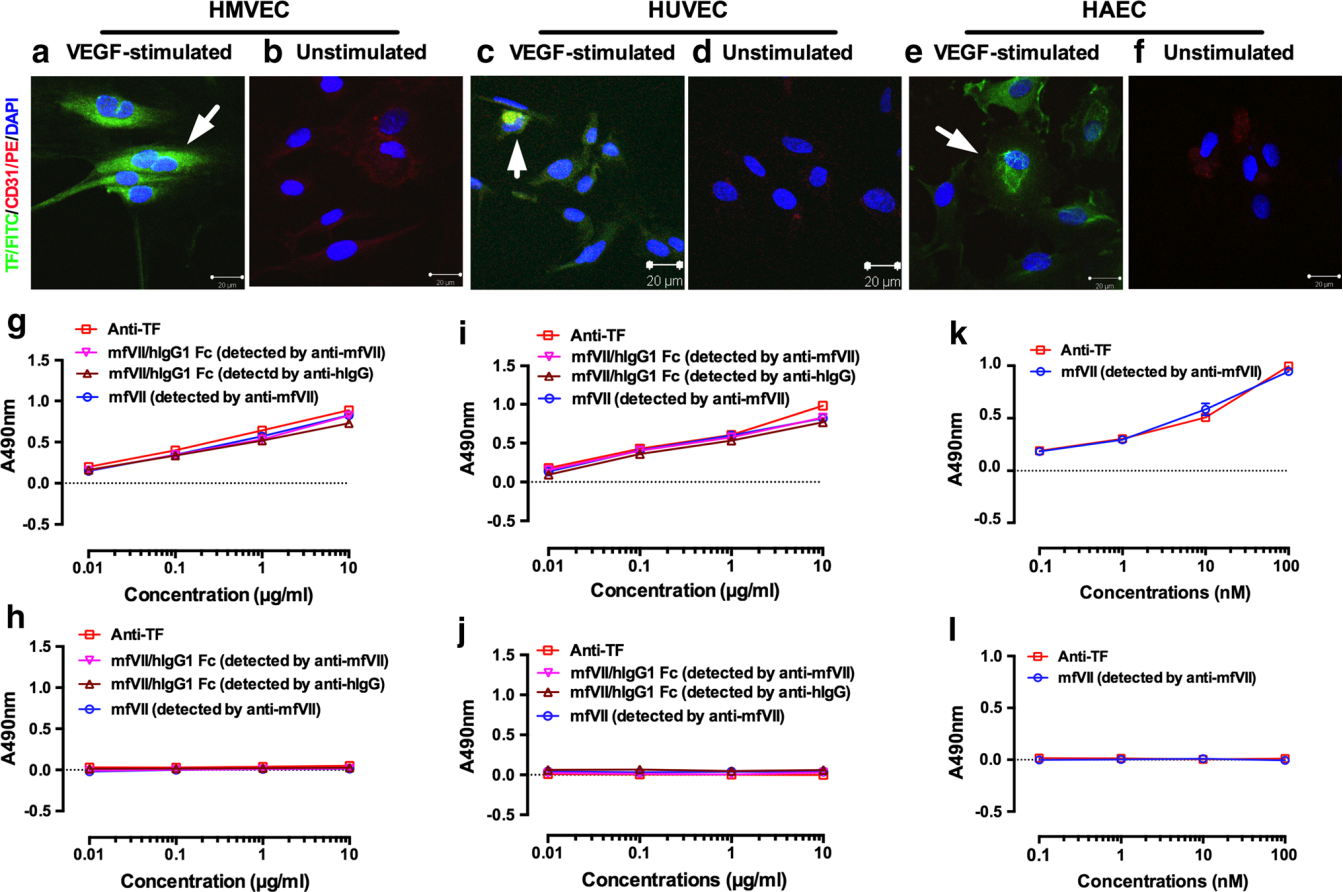
Selective binding of fVII agents to in vitro angiogenic VEC models. **a**–**f** Representative confocal imaging of different expression patterns of TF on in vitro angiogenic and quiescent VEC models. HMVEC (**a**, **b**), HUVEC (**c**, **d**) and HAEC (**e**, **f**). **g–l** Selective binding of fVII agents (mfVII/hIgG1 Fc and mfVII peptides) and anti-TF antibody to angiogenic VECs (**g**, **i**, **k**), but not to quiescent VECs (**h**, **j**, **l**). HMVEC (**g**, **h**), HUVEC (**i**, **j**) and HAEC (**k**, **l**). *Scale bars* in **a–f**: 20 μm. *p* values were calculated by 2-way ANOVA with multiple comparisons test. Data in **g–l** are presented as Mean ± SD and representative of two independent experiments

Next, we examined the binding (specificity) of fVII therapeutic agents to angiogenic VECs. To generate angiogenic VEC with maximal TF expression, VECs were incubated with 1 nM VEGF for 4 h based on the results in Fig. 1. Confocal imaging confirmed that TF was expressed on angiogenic HMVEC (Fig. 2a), HUVEC (Fig. 2c) and HAEC (Fig. 2e) after 4 h stimulation with VEGF, but not on resting VECs (Fig. 2b, d, f). Taken the results in Figs. 1 and 2, we conclude that TF is selectively expressed on angiogenic VEC, which can be induced on VECs from all three types of blood vessels (vein, microvessels and artery).

To assess the binding specificity of murine fVII/human IgG1 Fc (mouse ICON) and murine fVII/Sp (simplified as mfVII in Fig. 2) to angiogenic VECs, 4 h VEGF-stimulated VECs (Fig. 2g, i, k) and unstimulated VECs (Fig. 2h, i, j) were analyzed by cell ELISA. To ensure that cell membrane-bound mfVII/hIgG1 Fc was intact, we separately used anti-mfVII and antihuman IgG Fc antibody horseradish peroxidase conjugates for detection of mfVII/hIgG1 Fc in the cell ELISA. The cell ELISA results in Fig. 2g, h, i are summarized as follows. (1) A mouse monoclonal anti-TF antibody (clone HTF1) demonstrated that angiogenic VECs expressed TF (HMVEC in Fig. 2g, HUVEC in Fig. 2i and HAEC in Fig. 2k), whereas quiescent VECs do not express TF (Fig. 2h, j, l). The TF results by cell ELISA are consistent with the Western blotting results (Fig. 1) and confocal imaging results (Fig. 2a, b, c, d, e, f). (2) Similar to observations made with the anti-TF antibody, fVII agents, either in dimeric (mfVII/hIgG1 Fc) or monomeric form (mfVII), selectively bound all three angiogenic VEC types (Fig. 2g, i, k, respectively). In contrast, fVII agents did not bind to any of the quiescent VECs (Fig. 2h, 2j, l). Further, results in Fig. 2f, g suggested that mfVII/hIgG1 Fc was intact when binding to angiogenic HMVEC and HUVEC since there was no difference between anti-mfVII and anti-hIgG antibodies for detection of mfVII/hIgG1 Fc in cell ELISA. These findings established that (1) TF is selectively expressed on angiogenic VECs; (2) fVII agents selectively bind angiogenic VECs, likely reflecting their binding to TF (See Figs. 1 and 2).

**Fig. 3:**
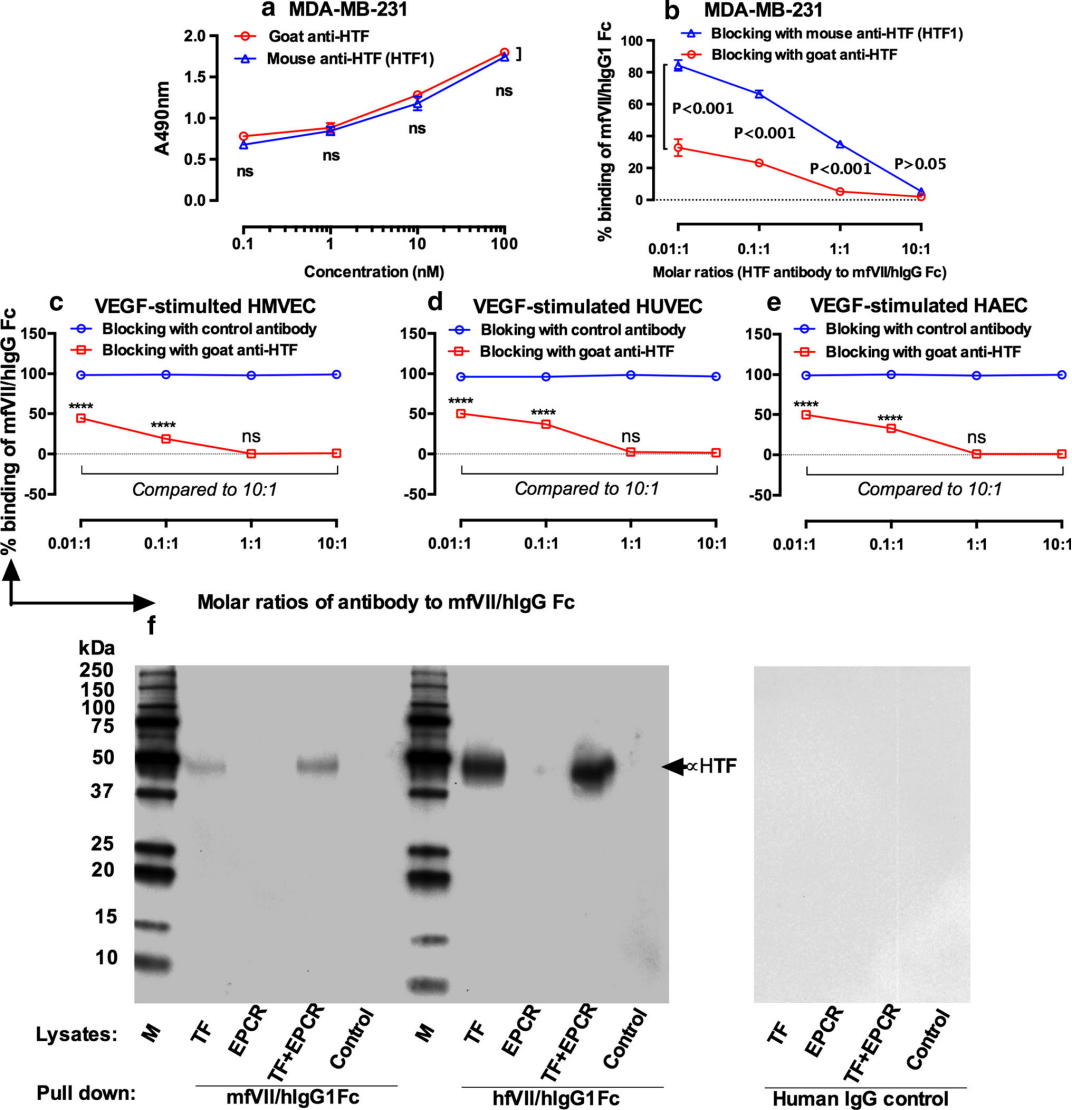
Selective binding of fVII agents to angiogenic VECs is mediated by TF. **a**–**b**. Choice of anti-TF antibodies for inhibiting the binding of fVII agent (mfVII/hIgG1 Fc) to TF. MDA-MB-231 is a human breast cancer line with high level of TF expression and is used here as a TF-expressing cell model. Two antihuman TF antibodies, goat anti-HTF and mouse anti-HTF (Clone HTF1), display similar binding to the MDA-MB-231 cells (**a**) but goat anti-TF antibody shows stronger inhibitory effect than monoclonal HTF1 in blocking mfVII/hIgG1 Fc binding to cancer cell TF (**b**). **c–e** Selective binding of mfVII/hIgG1 Fc to angiogenic HMVEC (**c**), HUVEC (**d**) and HAEC (**e**) can be completely blocked by goat anti-HTF, but not by control antibody. **f** Representative Western blots using mfVII/hIgG1 Fc and hfVII/hIgG1 Fc to immune-precipitate their cognate receptor TF (**f**). The negative control was the untransfected CHO-K1 cells. Human IgG was an isotype control. *p* values were calculated by 2-way ANOVA with multiple comparisons test. Data in **a**–**e** are presented as Mean ± SD and representative of two independent experiments

To confirm that selective binding of fVII agents to angiogenic VECs is mediated by TF, we carried out experiments that directly evaluated the ability of anti-TF antibodies to inhibit fVII binding to TF on angiogenic VECs. Two antihuman TF antibodies, mouse monoclonal antibody HTF1 and a goat polyclonal antibody (goat anti-HTF) (American Diagnostica), which were known to be able to block TF coagulation [29, 33], were compared for their relative inhibition of fVII binding to TF. Initial studies established that both anti-TF antibodies (*p* > 0.05) similarly bind to a breast cancer line (MDA-MB-231) that expresses high levels of TF [16] (Fig. 3a). Subsequently, goat anti-HTF was found to display a significantly stronger effect than HTF1 on inhibiting the binding of mfVII/hIgG1 Fc to MDA-MB-231 cancer cells (*p* < 0.001) (Fig. 3b). These observations suggested using goat anti-HTF as an inhibitory antibody for the experiments described in Fig. 3c, d, e.

To determine whether fVII is a ligand for TF on in vitro angiogenic VEC models, we developed an inhibitory cell ELISA assay in which angiogenic VEC were first incubated with various nanomolar concentrations of inhibitory goat anti-HTF for blocking TF or anti-EPCR (RCR-379) for blocking EPCR [30] as a control antibody, followed by incubation with 10 nM mfVII/hIgG1 Fc (to generate molar ratios 0.01:1–10:1, inhibitory antibody to mfVII/hIgG Fc). The binding of fVII/IgG1Fc was detected with anti-hIgG HRP conjugate. We found that goat anti-HTF completely (at ratios of 1:1 and 10:1) or partially (at ratios of 0.01:1 and 0.1:1) inhibit the binding of mfVII/hIgG1 Fc to all three angiogenic VECs (See Fig. 3c, d, e; HMVEC in Fig. 3c, HUVEC in Fig. 3d and HAEC in Fig. 3e). By contrast, control antibody did not inhibit fVII binding to angiogenic VECs, demonstrating that on angiogenic VECs, fVII acts via binding to TF. To further verify the specificity of fVII agents to TF versus the endothelial protein C receptor (EPCR) [34], we carried out immunoprecipitation–Western blotting (IP–WB) analysis using stable CHO-K1 cell lines expressing TF, EPCR, TF and EPCR and the control was untransfected CHO-K1 cells [35]. The results showed that mouse and human ICON could pull down TF efficiently in the presence and absence of EPCR (Fig. 3f), demonstrating that fVII agents (fVII/IgG1 Fc, ICON) specifically bind to TF.

**Fig. 4:**
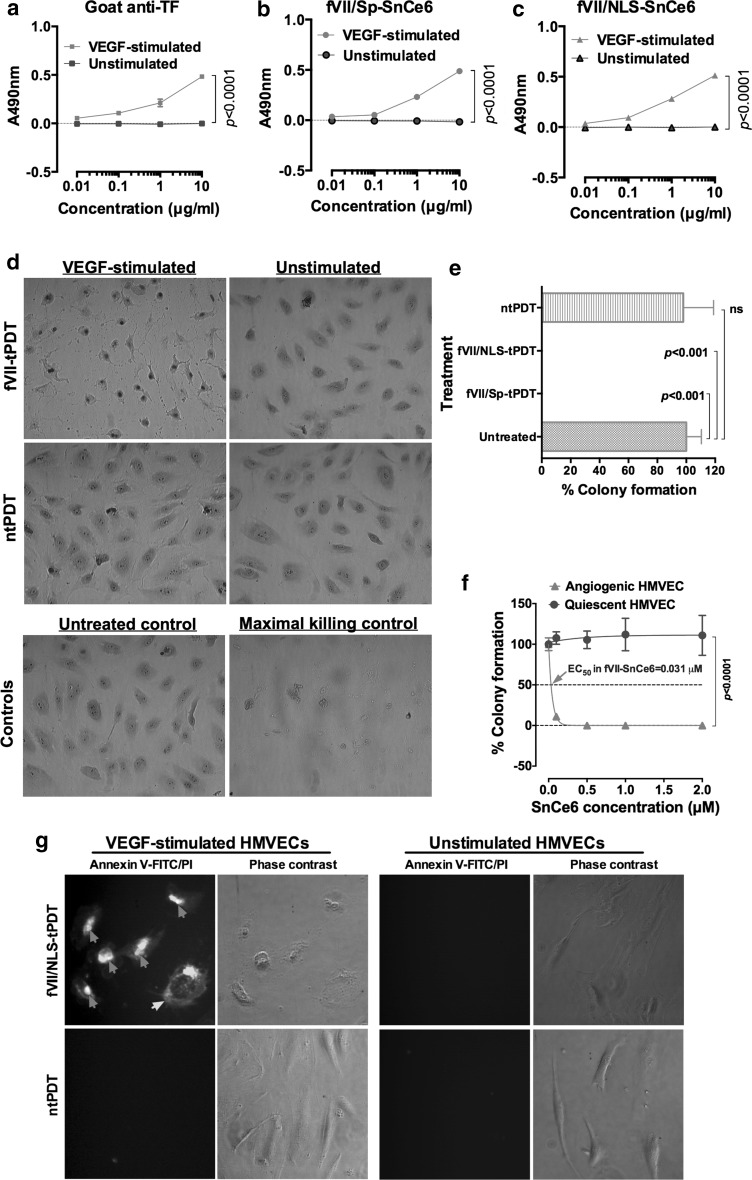
fVII-tPDT is selective and effective in eradicating angiogenic VEC. **a**–**c** fVII-SnCe6 conjugates retains the binding activity and selectivity to angiogenic VECs (HMVEC). Goat anti-TF was a positive control (**a**), SnCe6 was separately conjugated with mfVII/Sp (**b**) and mfVII/NLS (**c**) **d** Representative imaging of crystal *violet-stained* VEGF-stimulated and unstimulated HMVECs right after being treated with fVII-tPDT or ntPDT (2 μM and 635 nm laser light at 36 J/cm^2^). Control HMVECs include an untreated control and a maximal killing control (completely lysed by 1% Triton X-100). Original magnification: 400× phase contrast. **e** Complete eradication (no colonies formed) of angiogenic VEC (HMVEC) by fVII-tPDT using fVII/NLS-SnCe6 or fVII/Sp-SnCe6 (*p* < 0.001 vs. untreated control), whereas ntPDT has no therapeutic effect in killing angiogenic VEC under the same condition as for fVII-tPDT (2 μM SnCe6 and 635 nm laser light at 36 J/cm^2^). **f** The fVII-tPDT is effective and selective in killing angiogenic VEC (angiogenic HMVEC) with an EC_50_ of 0.031 μM SnCe6 in fVII/NLS-SnCe6, whereas it has no side effects to quiescent VEC (HMVEC). The fVII-tPDT conditions were as follows: 635 nm laser light at 36 J/cm^2^ and the SnCe6 concentrations in the fVII/NLS-SnCe6 conjugate (*x* axis) were 0.0 (buffer only), 0.5, 1 and 2 μM, respectively. Note that the VEC cells without fVII/NLS-SnCe6 (0.0 μM) also served as the light only control as they were also irradiated with 635 nm laser light (36 J/cm^2^). **g** The underlining mechanism of fVII/NLS-tPDT involves rapid induction of apoptosis and necrosis. SnCe6 concentration was reduced to 1 μM (36 J/cm^2^) so that not all of the treated cells undergo necrosis immediately after fVII-tPDT (See the stained HMVEC in **d**). Annexin V-FITC (*green*) stains for apoptotic cell membrane (*green arrow*) and propidium iodide (PI, *red*) stains for the nuclei of dead cells (*red arrows*). Original magnification: 400×. *p* values were calculated by 2-way ANOVA with multiple comparisons test. Data are presented as Mean ± SD and representative of two or three independent experiments. (Color figure online)

To determine whether fVII agents are selective and effective in killing angiogenic VECs, we tested fVII-tPDT using photosensitizer SnCe6-conjugated monomeric mfVII peptides. We first confirmed that like goat anti-TF antibody (Fig. 4a), PS-conjugated mfVII/Sp (Fig. 4b) and mfVII/NLS (Fig. 4c) selectively binds to angiogenic HMVECs. Then, we tested the effectiveness and selectivity of fVII-tPDT for in vitro killing of angiogenic HMVEC using the following assays:

1. The first assay was designed to observe immediate effect of fVII-tPDT by using crystal violet staining and photography under microscope for observation of cellular morphology overnight after fVII-tPDT treatment. We found that fVII-tPDT selectively and effectively killed angiogenic HMVEC (see VEGF-stimulated in Fig. 4d), whereas it had no obvious effects on unstimulated, quiescent HMVEC (normal VEC control). In contrast, nontargeted PDT (ntPDT) under the same conditions as fVII-tPDT except using free SnCe6 had no effect on killing angiogenic and quiescent HMVEC (Fig. 4d).
2. The second test for the selectivity and effectiveness of fVII-tPDT to angiogenic VECs was a clonogenic assay, which is designed for observing longer-term cell viability and proliferation. To target TF for photodynamic therapy, we made two mfVII peptides, namely mfVII/Sp and mfVII/NLS. Both are composed of single-chain peptide of mature murine factor VII with an active site mutation (K341A) followed by a 14-amino acid residue S tag (Sp) and a 13-aa nuclear localization sequence (NLS) [6, 15], respectively. We showed that two weeks after treatment with fVII-tPDT using mfVII-SnCe6 conjugates, angiogenic HMVEC did not form any colonies (See Fig. 4e), indicating that fVII-tPDT was able to completely eradicate angiogenic VECs. In contrast, ntPDT-treated angiogenic HMVEC formed similar numbers of colonies as untreated control cells (*p* value not significant).
3. Using the half-maximally effective concentration (EC_50_) as an indicator, based on clonogenic assay results, we found that the EC_50_ of SnCe6 in fVII-SnCe6 tPDT was 0.031 μM for angiogenic HMVEC, whereas it had no observable killing effect on normal quiescent VEC (see Fig. 4f). Taken together, the results in Fig. 4d, e, f suggested that the fVII-tPDT is selective and effective in eradicating angiogenic VECs.

To better understand its mechanism of action of fVII-tPDT, we first treated angiogenic and resting HMVEC with fVII-tPDT (mfVII/NLS-SnCe6) or ntPDT (SnCe6) then immediately tested the VEC for apoptosis using Annexin V-FITC and for necrosis using propidium iodide (PI). We found that fVII-tPDT treatment induced immediate apoptosis and necrosis in angiogenic HMVECs (see Fig. 4g), similar to its mechanism in killing human lung cancer cells [6]. Importantly, fVII-tPDT did not induce detectable apoptosis and necrosis in quiescent HMVEC, whereas ntPDT had no effect in angiogenic and quiescent HMVEC (Fig. 4g).

## Discussion

Identification of specific anti-angiogenic target molecules in endothelial is crucial in developing anti-angiogenesis therapies. Toward this end, a major effort has focused on targeting VEGF and VEGFRs. Elevated levels of VEGFRs are detected on tumor VECs in many types of solid cancers [36]. Unfortunately, VEGFRs are not angiogenic-specific endothelial receptors since they are also expressed on normal endothelium in many organs [37, 38]. Nevertheless, this study defines the peak expression of TF on angiogenic VECs (4 h VEGF induction), which may be used as an optimized time point for generating in vitro VEC models for identification of more novel target molecules by revealing the differences in gene expression (for example, mRNAs and micro-RNAs) between angiogenic and quiescent VECs.

In this report, we show that, unlike VEGFRs, TF is a specific receptor for angiogenic VECs in vitro. It appears that TF is also a unique pathological angiogenic endothelial cell surface receptor in vivo because of its selective expression on angiogenic VECs in vivo in tumor vasculature [4–7, 9, 14], ocular [18] and endometriotic [21] neovasculature from patients or animal models. In addition to TF on angiogenic VEC, TF is overexpressed on many cancer cells including solid cancer and leukemia [39]. Therefore, TF appears to be a common yet specific biomarker and target molecule for both the pathological neovasculature and cancer cells [6, 7, 16, 39].

Consequently, the use of fVII as targeting vehicle enabled our group to develop two TF-targeted therapeutics, namely fVII/IgG1 Fc (ICON) for immunotherapy and fVII/PS for tPDT, which display selectivity, effectiveness and safety in the treatment of lung and breast cancer (regardless of their drug resistance and other membrane targets) [5–7, 13–17, 23], macular degeneration [18–20] and/or endometriosis [21] in preclinical mouse, rat and pig models and in phase I and II clinical trials for AMD patients (NCT01485588) [22]. In this study, we showed that both mfVII/IgG1 Fc and hfVII/IgG1 Fc bind to human TF, which is only expressed by angiogenic VECs upon VEGF stimulation. Thus, TF-targeted ICON and fVII-tPDT should not cause side effects to quiescent vessels, including microvessels, venous and arterial vessels, while they can selectively target and effectively eradicate angiogenic VECs via TF targeting. Nevertheless, caution should be taken as VEGF and other chemokines (IL-1β, TNF-α, etc.) may potentially induce TF expression on normal resting VECs. They should be monitored before and during the treatment of fVII agents, particularly when fVII agents are administered systemically. In the case of cancer, Drs. Yang and Rosenberg et al. [40] reported that 76 of 113 patients with metastatic renal cell carcinoma had a baseline VEGF level below the lower limit of detection (40 pg/ml). After anti-VEGF antibody bevacizumab (Avastin) therapy, the plasma VEGF levels increased to a range of 150–250 pg/ml. Similarly, a very recent study [41] reported that the plasma baseline levels of VEGF in 60 patients (Hongkong) with colorectal cancer metastases were about 10 pg/ml, and they were increased to 40 pg/ml after anti-VEGF antibody bevacizumab (Avastin) therapy [41]. Importantly, the group found that increased VEGF was in a complex with the neutralizing antibody that prevents VEGF from binding to VEGF receptors. In the cases of AMD and PCV (polypoidal choroidal vasculopathy), Tong et al. [42] reported that the aqueous humor level of VEGF was about 400 pg/ml in eyes with PCV and about 700 pg/ml in eyes with choroidal neovascularization of AMD. A Japanese group also reported aqueous humor level of VEGF was higher in AMD and PCV than control eyes. But they did not find the significant difference of VEGF levels between AMD and PCV. Nevertheless, the levels of VEGF reported in those published studies were at 100-fold lower than the concentration (1 nM VEGF = 38 ng/ml, MW 38 kDa) that we tested in this study. It remains to investigate the lowest VEGF concentration that is able to induce endothelial TF expression. If the blood VEGF level in patients is detected high (enough to induce TF expression on normal endothelial cells), anti-VEGF inhibitors may be administered to neutralize and clear VEGF from circulation, if any, prior to fVII-targeted therapies. Regardless of the VEGF levels in AMD and PCV, fVII-tPDT may be used for the treatment of these ocular diseases as long as TF expression is detected in the neovascularization lesions.

In conclusion, this study identifies that TF is an angiogenic-specific endothelial biomarker and verifies that TF is the therapeutic target specific for fVII agents. This study also elucidated for the first time that the specificity of fVII agents for angiogenic VEC is mediated via binding TF. Thus, this study not only identifies TF as an angiogenic endothelial receptor specific for fVII agents, but also helps predict therapeutic efficacy and potential side effects of these fVII-targeted agents. As such, supports use of TF-targeted therapeutics in clinical trials of several angiogenesis-dependent common human diseases, notably cancer, macular degeneration, endometriosis and rheumatoid arthritis, in which VEGF plays a central role in pathological angiogenesis.
